# The mediating role of activities of daily living in the association between nutritional risk and cognitive function among stroke survivors: a cross-sectional study

**DOI:** 10.3389/fmed.2026.1899617

**Published:** 2026-08-20

**Authors:** Lei Bin, Fang Han, Wanhong Luo, Minghui Yong, Mingyu Wang, Xia Xie

**Affiliations:** 1 School of Nursing, North Sichuan Medical College, Nanchong, China; 2 Department of Rehabilitation, Affiliated Hospital of North Sichuan Medical College, Nanchong, China

**Keywords:** activities of daily living, cognitive function, mediation analysis, nutritional risk, stroke

## Abstract

**Background:**

Nutritional risk is linked to poorer cognitive function in stroke patients, but whether this association is mediated by other factors remains unclear. The purpose of this study is to examine the relationship between nutritional risk and cognitive function, and to evaluate whether activities of daily living (ADL) mediate this association.

**Methods:**

A cross-sectional design with convenience sampling was employed to enroll stroke patients from a tertiary hospital in Sichuan, China (January–June 2025). Data were collected using the Nutritional Risk Screening 2002, the Barthel Index, and the Mini-Mental State Examination. Mediation analysis was performed using bootstrap methods, adjusting for sociodemographic and clinical characteristics. Sensitivity analyses were conducted using the E-value and sequential ignorability to test the robustness of the mediating effect.

**Results:**

A total of 472 stroke patients were enrolled with a mean age of 66.99 ± 11.52 years. The mean scores were 2.68 ± 0.99 for nutritional risk, 60.35 ± 20.47 for activities of daily living, and 19.08 ± 7.65 for cognitive function. After adjusting for covariates, the direct effect of nutritional risk on cognitive function was −1.40 (95% CI: −2.231 to −0.539; *p* = 0.001). An indirect effect mediated by ADL was observed (*β* = −0.44, 95% CI: −0.870 to −0.039; *p* = 0.040), which accounted for 24.0% of the total effect. Sensitivity analyses yielded an E-value of 1.29 for the indirect effect, with a critical *ρ* of 0.4 required to nullify the mediation.

**Conclusion:**

ADL partially mediates the association between nutritional risk and cognitive function in stroke survivors. Future prospective studies are needed to evaluate whether interventions targeting ADL are associated with improved cognitive outcomes.

## Introduction

Cognitive dysfunction is a common complication after stroke, with an incidence reported as high as 83% (1). It not only impairs patients’ rehabilitation, limits their social participation, and reduces their quality of life (2–4), but also leads to many patients eventually developing dementia (5). Therefore, identifying modifiable risk factors for cognitive decline in stroke survivors is critical, especially as the global population ages and their numbers increase.

Nutritional status is an important modifiable factor associated with stroke prognosis (6). Stroke patients often face nutritional risk due to dysphagia, reduced appetite, and metabolic disorders (7). Recent studies have shown that nutritional risk is associated with cognitive decline, with multiple biological pathways proposed to underlie this relationship, including deficits in neurotransmitter synthesis, oxidative stress, and altered brain-derived neurotrophic factor levels (8–10). However, these mechanisms do not fully explain the relationship. Nutritional risk may be related to cognition indirectly through behavioral or functional factors, but whether such factors mediate this association remains unclear.

Activities of daily living (ADL) are a core indicator of functional status and a key goal of stroke rehabilitation. Studies have shown that ADL are associated with both nutritional status and cognitive function (11–13). On one hand, nutritional risk is associated with reduced muscle mass and strength, which are in turn associated with impaired motor function and daily activities (14). On the other hand, loss of independence in ADL may be associated with cognitive decline, and this association may be related to reduced social participation and increased psychological stress (15). Existing literature reports that nutritional risk is associated with cognitive function and ADL, and that ADL is also related to cognitive function (6, 16, 17). These lines of evidence are consistent with the hypothesis that ADL may mediate the association between nutritional risk and cognitive function.

However, most existing evidence is limited to pairwise associations, with few studies integrating nutritional risk, ADL, and cognitive function within a mediation framework. Consequently, the mediating role of ADL in the relationship between nutritional risk and cognitive function remains unknown. To address this gap, we conducted a cross-sectional study among stroke patients to examine whether and to what extent ADL mediate this association.

## Methods

### Ethical approval and informed consent

The Ethics Committee of the participating hospital reviewed and approved the study (Approval No.: 2025ER585-1). Written informed consent was obtained from all participants. Participant confidentiality was maintained throughout the study, and all data were anonymized and restricted to statistical purposes only.

### Study participants

This study employed a cross-sectional approach with convenience sampling to enroll stroke patients admitted to a tertiary hospital in Sichuan, China, between January and June 2025. The inclusion criteria were: (1) stroke diagnosis verified by computed tomography or magnetic resonance imaging per World Health Organization standards (18); (2) age ≥ 18 years; (3) adequate consciousness to complete all evaluations; and (4) signed informed consent. Patients were excluded if they had: (1) severe organ dysfunction (e.g., cardiac, respiratory, renal failure); (2) comorbid neurological disorders (e.g., dementia, epilepsy, Parkinson’s disease); or (3) ongoing involvement in other interventional trials.

Sample size determination was performed using G*Power 3.1. The analysis assumed a medium effect size (f^2^ = 0.15), with a significance level of 0.05 and statistical power of 0.80. The number of predictor variables ranged from 2 to 15 depending on the analysis, giving a minimum sample size of 68 to 145. Because mediation analysis with bootstrap methods requires larger samples, the target sample size was set above 200 following recommendations from Fritz and MacKinnon (19). Considering a potential 20% invalid response rate, the targeted sample size was set at 250.

Of the 538 stroke patients initially assessed, 500 met the inclusion criteria and were invited to participate. The remaining 38 patients were excluded due to severe organ dysfunction (*n* = 4), comorbid neurological disorders (*n* = 5), ongoing involvement in other interventional trials (*n* = 26), or refusal to participate (*n* = 3). Among the 500 distributed questionnaires, 28 were excluded due to incomplete responses or patterned answering, and 472 valid questionnaires were included in the final analysis.

### Data collection

Trained investigators administered one-on-one survey within the ward setting. Before data collection, we explained the study’s purpose, significance, and confidentiality procedures patients and their families and obtained written consent. Patients completed the questionnaire independently; for those unable due to literacy or physical limitations, investigators read each item and recorded responses. Questionnaires were checked for completeness upon collection, and omissions were addressed with participants when feasible. Questionnaires that remained incomplete or showed patterned response were excluded, and no missing data imputation was performed. All analyses were therefore based on complete cases.

### Assessment of sociodemographic and clinical variables

Sociodemographic and clinical information was collected using a self-designed structured questionnaire developed by the research team based on relevant literature and clinical practice. Variables included age, gender, education, marital status, body mass index (BMI), stroke subtype, disease duration, history of stroke, number of comorbidities, and lifestyle factors (smoking and drinking). Swallowing function was screened using the Eating Assessment Tool-10 (EAT-10), where a score of 3 or above indicated a potential risk for dysphagia.

### Evaluation of cognitive function

We employed the Mini-Mental State Examination (MMSE) (20) to evaluate participants’ cognitive function. The MMSE was chosen for this hospitalized stroke population due to its good performance in individuals with lower educational backgrounds and its primarily verbal format, which minimizes the impact of motor impairments on cognitive assessment (5). This 11-item scale covers seven dimensions, namely orientation, registration, attention and calculation, recall, and language. Total scores range from 0 to 30, where higher values reflect superior cognitive performance.

### Screening of nutritional risk

Nutritional risk was evaluated using the Nutritional Risk Screening 2002 (NRS 2002) (21). The tool consists of three parts: nutritional status (scored 0–3), disease severity (scored 0–3), and an age correction (1 point if aged ≥70 years). Summed scores range from 0 to 7, wherein higher scores reflect increased nutritional risk. This instrument demonstrates satisfactory psychometric properties for hospitalized individuals (22).

### Assessment of activities of daily living

The Barthel Index (BI) (23) was applied to measure ADL. This index assesses independence across 10 key daily tasks, including feeding, personal hygiene (bathing and grooming), dressing, continence (bladder and bowel management), toilet use, transfers (bed-to-chair), mobility (ambulation), and stair climbing. The total score ranges from 0 to 100, where higher scores indicate greater independence. The BI is widely used in stroke rehabilitation with good reliability and validity (24).

### Statistical analysis

We conducted all statistical analyses with SPSS (version 27.0) and R (version 4.5.0). Categorical data were expressed as frequencies and percentages, while continuous data were presented as means ± standard deviations. Given the relatively large sample size (*n* = 472) and the well-documented robustness of parametric tests to violations of normality (25), parametric methods were employed for univariate analyses. Specifically, independent *t*-tests were used to compare groups regarding nutritional risk, ADL, and cognitive function, and Pearson correlation was used to examine unadjusted associations among age, BMI, and the three primary variables. Mediation analysis used the R mediation package, with unadjusted and covariate-adjusted models. Covariates in the adjusted model were baseline characteristics significantly associated with any of the three main variables in univariate analyses. Multicollinearity among these variables was assessed using variance inflation factor, with all values below 5. The indirect effect’s 95% confidence interval (CI) was constructed through bias-corrected bootstrapping based on 5,000 resamples, with significance determined by a CI not containing zero. To assess robustness to unmeasured confounding, we calculated the E-value, defined as the minimum association strength needed to nullify the indirect effect. Larger E-values indicate lower sensitivity to unmeasured confounding. We also performed sequential ignorability sensitivity analysis, examining the error correlation *ρ*; larger absolute critical ρ indicates more robust finding. All statistical tests were two-tailed with *α* = 0.05.

## Results

### Participant characteristics

This study included 472 stroke patients. The average age was 66.99 years (SD = 11.52) with males accounting for 62.08% of the sample. The majority (79.45%) had completed junior high school education or less. Ischemic stroke accounted for 81.99% of cases, and 75% had disease duration ≤6 months. Other baseline characteristics were presented in Table 1.

**Table 1:** Baseline characteristics of participants and subgroup comparisons of NRS2002, Barthel Index, and MMSE scores (*n* = 472).

Variables	Total *n* (%)	NRS 2002		BI		MMSE	
		M ± SD	*P*	M ± SD	*P*	M ± SD	*P*
Gender			0.001		0.708		<0.001
Male	293 (62.08)	2.57 ± 0.93		60.62 ± 20.89		20.17 ± 7.30	
Female	179 (37.92)	2.87 ± 1.05		59.89 ± 19.82		17.30 ± 7.89	
Education level			<0.001		0.258		<0.001
Junior high school and below	375 (79.45)	2.77 ± 0.98		59.80 ± 20.05		18.28 ± 7.56	
Senior high school and above	97 (20.55)	2.37 ± 0.95		62.44 ± 22.03		22.18 ± 7.23	
Marital status			0.005		0.285		0.058
Married	429 (90.89)	2.63 ± 0.94		60.66 ± 20.12		19.29 ± 7.57	
Single	43 (9.11)	3.21 ± 1.26		57.16 ± 23.73		16.98 ± 8.15	
Number of comorbidities			0.342		0.422		0.107
<Three disease	295 (62.50)	2.65 ± 0.99		60.93 ± 20.47		19.52 ± 7.57	
≥Three disease	177 (37.50)	2.74 ± 0.98		59.37 ± 20.50		18.35 ± 7.75	
Type of stroke			0.007		<0.001		0.863
Ischemic stroke	387 (81.99)	2.74 ± 0.97		62.79 ± 18.96		19.11 ± 7.69	
Hemorrhagic stroke	85 (18.01)	2.42 ± 1.00		49.20 ± 23.35		18.95 ± 7.50	
History of stroke			0.012		0.003		0.668
First-ever	366 (77.54)	2.62 ± 0.97		58.83 ± 20.78		19.16 ± 7.69	
Recurrent	106 (22.46)	2.90 ± 1.01		65.59 ± 18.51		18.80 ± 7.52	
Duration of stroke			0.501		0.004		0.576
≤6 months	354 (75.00)	2.67 ± 0.98		58.78 ± 20.59		18.97 ± 7.66	
>6 months	118 (25.00)	2.74 ± 1.01		65.03 ± 19.46		19.42 ± 7.63	
EAT-10			0.153		<0.001		0.021
<3	315 (66.74)	2.73 ± 1.01		62.95 ± 18.57		19.65 ± 7.49	
≥3	157 (33.26)	2.59 ± 0.94		55.13 ± 23.03		17.94 ± 7.87	
Smoking			0.027		0.522		0.145
No	303 (64.19)	2.76 ± 0.99		59.89 ± 20.04		18.70 ± 7.86	
Yes	169 (35.81)	2.55 ± 0.98		61.15 ± 21.26		19.77 ± 7.22	
Drinking			<0.001		0.991		<0.001
No	195 (41.31)	2.87 ± 0.98		60.33 ± 20.17		17.46 ± 7.89	
Yes	277 (58.69)	2.56 ± 0.98		60.35 ± 20.72		20.23 ± 7.27	

M ± SD: mean ± standard deviation.

### Nutritional risk, activities of daily living and cognitive function scores

The mean scores were 2.68 ± 0.99 for nutritional risk, 60.35 ± 20.47 for ADL, and 19.08 ± 7.65 for cognitive function (Table 2).

**Table 2:** Means, standard deviations, and correlations among age, BMI, nutritional risk, activities of daily living, and cognitive function (*n* = 472).

Variables	M ± SD	1	2	3	4	5
1. Age	66.99 ± 11.52	–				
2. BMI (kg/m^2^)	23.98 ± 3.37	−0.211***	–			
3. Nutritional risk	2.68 ± 0.99	0.642***	−0.519***	–		
4. Activities of daily living	60.35 ± 20.47	−0.088	0.100**	−0.155***	–	
5. Cognitive function	19.08 ± 7.65	−0.302***	0.148*	−0.339***	0.441***	–

* *P* < 0.05; ** *P* < 0.01; *** *P* < 0.001; M ± SD: mean ± standard deviation.

### Comparison of nutritional risk, activities of daily living and cognitive function scores by participant characteristics

As shown in Table 1, nutritional risk scores differed significantly by gender, education, marital status, stroke type, history of stroke, smoking, and drinking; ADL scores by stroke type, history of stroke, EAT-10 score, and disease duration; and cognitive function scores by gender, education, EAT-10 score, and drinking (all *p* < 0.05).

### Correlations among continuous variables

As shown in Table 2, higher nutritional risk scores were associated with lower ADL (*r* = −0.155, *p* < 0.001) and poorer cognitive function (*r* = −0.339, *p* < 0.001). Better ADL performance was correlated with better cognitive function (*r* = 0.441, *p* < 0.001). Age was positively correlated with nutritional risk (*r* = 0.642, *p* < 0.001) and negatively with cognitive function (*r* = −0.302, *p* < 0.001). BMI was negatively correlated with nutritional risk (*r* = −0.519, *p* < 0.001) and positively with ADL (*r* = 0.100, *p* = 0.033).

### Mediating role of activities of daily living

**Figure 1:**
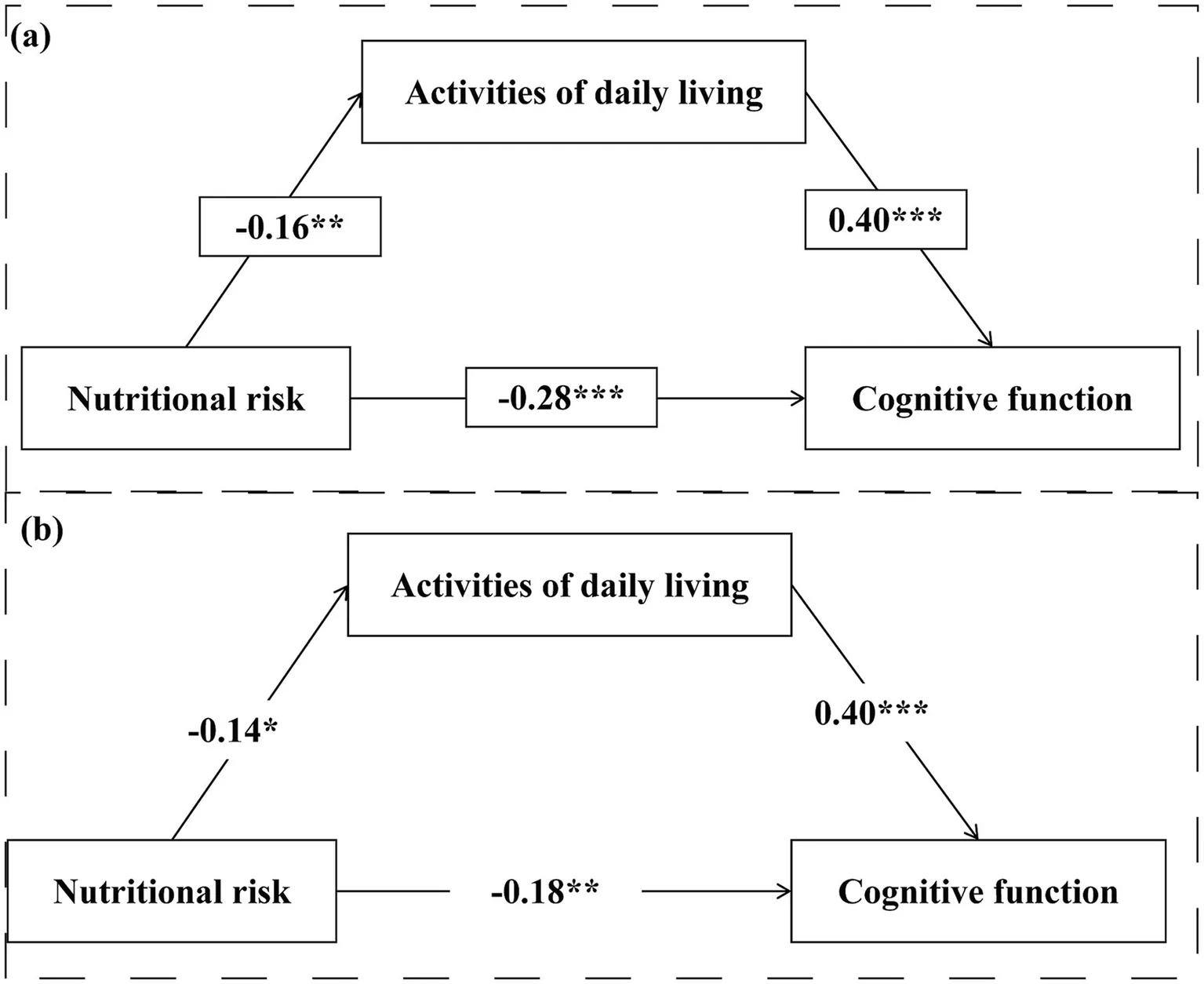
Standardized path coefficients for the mediation models. **(a)** Unadjusted model. **(b)** Model adjusted for age, gender, education level, marital status, stroke type, disease duration, history of stroke, EAT-10 score, smoking, and drinking. *** *p* < 0.001; ** *p* < 0.01; * *p* < 0.05.

The mediation analysis results are presented in Table 3 and Figure 1. In the unadjusted model, the total effect of nutritional risk on cognitive function was −2.63 (95% CI: −3.273 to −2.011). This total effect comprised a direct effect of −2.15 (95% CI: −2.719 to −1.579) and an indirect effect through ADL of −0.48 (95% CI: −0.801 to −0.218). The indirect effect accounted for 18.3% of the total effect. After adjustment for covariates, the indirect effect was −0.44 (95% CI: −0.870 to −0.039), with the proportion mediated increasing to 24.0%. The adjusted total and direct effects were −1.83 (95% CI: −2.704 to −0.936) and −1.40 (95% CI: −2.231 to −0.539), respectively.

**Table 3:** Mediation analysis of activities of daily living in the relationship between nutritional risk and cognitive function.

Effect	Model	b	SE	95% CI	*P*	E-value
Direct effect	Unadjusted	−2.15	0.29	−2.719, −1.579	<0.001	–
	Adjusted*	−1.40	0.43	−2.231, −0.539	0.001	–
Indirect effect	Unadjusted	−0.48	0.15	−0.801, −0.218	0.001	1.31 [1.177, 1.419]
	Adjusted*	−0.44	0.21	−0.870, −0.039	0.040	1.29 [1.052, 1.450]
Total effect	Unadjusted	−2.63	0.32	−3.273, −2.011	<0.001	–
	Adjusted*	−1.83	0.45	−2.704, −0.936	<0.001	–

b, unstandardized regression coefficient; SE, standard error; CI, confidence interval. The indirect effect was estimated using bias-corrected bootstrap with 5,000 resamples. The E-value for the indirect effect is shown with its 95% CI in brackets. * The adjusted model controlled for age, gender, education level, marital status, stroke type, disease duration, history of stroke, EAT-10 score, smoking, and drinking.

### Sensitivity analysis results

**Figure 2:**
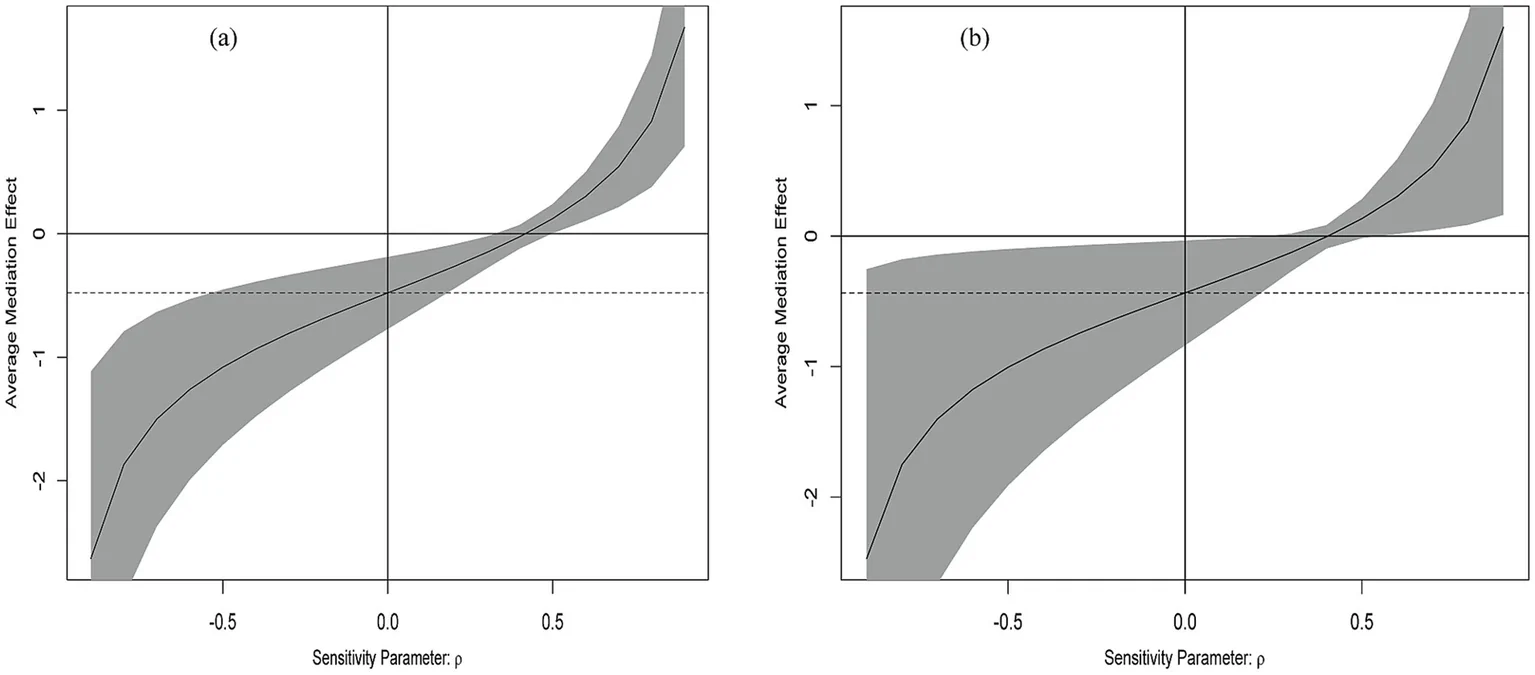
Sensitivity analysis for the mediation effect under sequential ignorability. **(a)** Unadjusted model. **(b)** Model adjusted for age, gender, education level, marital status, stroke type, disease duration, history of stroke, EAT-10 score, smoking, and drinking. *ρ* = error correlation coefficient between the mediator and outcome residuals under the sequential ignorability assumption. The shaded area represents the 95% confidence interval.

The E-value for the indirect was 1.31 (95% CI lower bound 1.18) for the unadjusted model and 1.29 (95% CI lower bound 1.05) for the adjusted model. Sequential ignorability analysis showed that the 95% CI for the average causal mediation effect first included zero when the error correlation *ρ* reached 0.4 in both models, with critical residual correlations of 0.16 and 0.09 for the unadjusted and adjusted models, respectively (Figure 2).

## Discussion

In this study, higher nutritional risk is linked to poorer cognitive function in patients with stroke. Mediation analysis revealed that ADL partially mediated this relationship, with a mediation proportion of 24.0%. The mediation remained significant after adjusting for multiple potential confounders, and sensitivity analyses were performed to further examine the robustness of the findings. The generalizability of these findings is limited by the single-center convenience sample, and the sample was predominantly composed of patients with ischemic stroke, early-stage disease, and relatively low educational attainment. These characteristics should be considered when extrapolating the results to other populations or settings.

Consistent with recent meta-analytic evidence consolidating this link in post-stroke populations (26), we observed an inverse relationship of nutritional risk with cognitive function, yet the effect size in our study is larger than that observed in general community-dwelling older adults (6). This may be because our participants were hospitalized stroke patients, who have a higher prevalence of nutritional risk (27), amplifying the impact on cognition. Biologically, potential explanations for this association include deficits in neurotransmitter production, oxidative damage, and lower concentrations of brain-derived neurotrophic factor (9). Notably, the direct effect of nutritional risk on cognition remained significant after covariate adjustment, indicating that this association is not fully explained by demographic or clinical characteristics. This highlights nutritional status as an independent correlate and supports routine screening, though prospective studies are warranted to confirm intervention benefits.

A notable finding of this study is the statistical indication of ADL as a potential partial mediator in the observed relationship. The biological plausibility of this indirect pathway is supported by prior evidence. In stroke patients at nutritional risk, inadequate protein intake reduces skeletal muscle protein synthesis (28). This reduction, coupled with post-stroke disuse atrophy, increases the risk of sarcopenia (29). Sarcopenia is known to directly impair basic activities such as transfer, walking, and toileting (30). This finding is consistent with the rehabilitation principle that nutritional support contributes to functional recovery (31). The association of ADL with cognitive function was stronger than the direct association between nutritional risk and cognition, suggesting that ADL may be more closely associated with cognitive function in this sample. Decline in ADL is correlated with reduced independence, less social participation, and fewer cognitive stimuli (32). Additionally, activity limitations may trigger negative emotions (e.g., anxiety, depression) which have been linked to cognitive decline through psychological stress (33, 34). Overall, these results are consistent with the “use it or lose it” theory of cognitive aging and underscore the potential relevance of functional training (35). Previous intervention studies have shown that ADL-oriented training or combined rehabilitation with nutritional support can lead to concurrent improvements in both functional and cognitive outcomes (36, 37), but none have formally quantified the mediating pathway proposed here. The indirect effect we observed therefore warrants further confirmation, preferably through longitudinal designs or trials specifically powered to test this mediation mechanism.

After adjusting for covariates, the proportion mediated increased from 18.3 to 24.0%, while the absolute direct effect decreased from 2.15 to 1.40 and the indirect effect showed minimal change. It indicates that covariates primarily associated with the direct pathway. The indirect effect of nutritional risk on cognition through ADL was −0.44 on the MMSE scale, with a relatively wide confidence interval whose upper bound was −0.039, indicating limited precision. Although the indirect effect remained detectable after covariate adjustment, the modest 0.44-point difference on the MMSE, combined with the cross-sectional design, suggests that its clinical relevance remains uncertain. Future studies are warranted to determine whether this indirect association translates into clinically meaningful cognitive changes.

We further assessed the robustness of the indirect effect to unmeasured confounding using two sensitivity analyses. The E-value of 1.29 implies that an unmeasured confounder with a risk ratio of 1.29 for both ADL and cognition would suffice to fully explain away the observed indirect effect. The critical *ρ* of 0.4 suggests that a moderate correlational strength of confounding would be required to nullify this effect. Collectively, these estimates indicate that the indirect effect shows limited robustness to unmeasured confounding. Therefore, these findings should be interpreted with caution, and uncollected factors such as stroke severity, depressive symptoms, and lesion characteristics could have confounded the estimated indirect effect. Future studies that include these measures would be valuable in determining whether the indirect pathway remains after more comprehensive confounder adjustment.

### Implications for clinical practice

The indirect association identified in this study suggests that ADL may be a relevant clinical indicator in the relationship between nutritional risk and cognitive function. This finding implies that cognitive preservation strategies should extend beyond nutritional supplementation alone to include functional maintenance and rehabilitation training, specifically by targeting ADL capacity to enhance the cognitive benefits derived from nutritional interventions. However, this finding warrants cautious interpretation given the cross-sectional design and the limited robustness to unmeasured confounding. Future research is needed to confirm these findings.

### Limitations

This study has several limitations. First, the cross-sectional design precludes causal inferences regarding the mediating pathway, longitudinal studies are needed to establish temporal sequences and confirm the directionality of these associations. Second, most participants in our sample were in the early post-stroke phase and had relatively low educational levels. These characteristics, together with the use of convenience sampling from a single tertiary hospital, may limit the generalizability of the findings. Third, the E-value of 1.29 indicates limited robustness to unmeasured confounding. Although the critical *ρ* of 0.4 suggests that moderate confounding would be required to nullify the effect, unmeasured factors such as stroke severity, lesion characteristics, pre-stroke functional status, rehabilitation intensity, social support, and depressive symptoms could still have influenced the estimates. Additionally, while the MMSE is widely used in stroke populations, it has been reported to be less sensitive than the MoCA for detecting vascular cognitive impairment and executive dysfunction. This may have led to an underestimation of cognitive impairment in some participants, particularly those with mild or predominantly executive deficits, and could have underestimated the observed associations. Future studies using the MoCA or domain-specific neuropsychological batteries are needed to further examine the robustness of our findings.

## Conclusion

The relationship between nutritional risk and cognitive function in individuals with stroke is partially mediated by ADL. From a clinical perspective, integrating nutritional screening with functional assessment and implementing early rehabilitation training to preserve ADL may be relevant factors to consider in this clinical care of this population. However, given the cross-sectional design, these findings warrant further examination in prospective studies.

## Data Availability

The raw data supporting the conclusions of this article will be made available by the authors, without undue reservation.
